# Shortcuts to adiabaticity by counterdiabatic driving for trapped-ion displacement in phase space

**DOI:** 10.1038/ncomms12999

**Published:** 2016-09-27

**Authors:** Shuoming An, Dingshun Lv, Adolfo del Campo, Kihwan Kim

**Affiliations:** 1 Center for Quantum Information, Institute for Interdisciplinary Information Sciences, Tsinghua University, Beijing 100084, China; 2 Department of Physics, University of Massachusetts, Boston, Massachusetts 02125, USA

## Abstract

The application of adiabatic protocols in quantum technologies is severely limited by environmental sources of noise and decoherence. Shortcuts to adiabaticity by counterdiabatic driving constitute a powerful alternative that speed up time-evolution while mimicking adiabatic dynamics. Here we report the experimental implementation of counterdiabatic driving in a continuous variable system, a shortcut to the adiabatic transport of a trapped ion in phase space. The resulting dynamics is equivalent to a ‘fast-motion video' of the adiabatic trajectory. The robustness of this protocol is shown to surpass that of competing schemes based on classical local controls and Fourier optimization methods. Our results demonstrate that shortcuts to adiabaticity provide a robust speedup of quantum protocols of wide applicability in quantum technologies.

## Abstract (web summary)

The application of adiabatic protocols in quantum technologies is limited due to the detrimental action of decoherence. Here the authors demonstrate a shortcut to adiabaticity via counterdiabatic driving in a trapped ion system.

## Graphical abstract

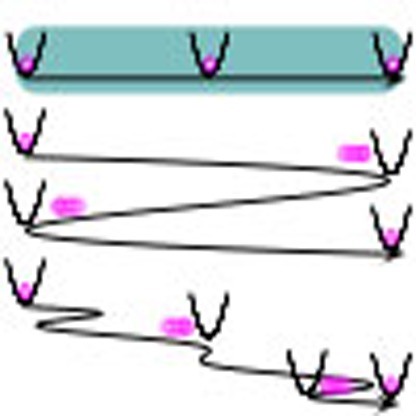

Adiabatic processes play an essential role in many aspects of quantum technology^1^,^2^. Quantum adiabatic simulation exploits adiabatic dynamics to track ground states of complex Hamiltonians facilitating the study of quantum many-body phenomena^3^,^4^. Schemes for scalable ion-trap quantum computer resort to the adiabatic transfer of ions between different trap zones^5^,^6^. Adiabatic dynamics plays as well a key role in holonomic quantum computation^7^, and the design of the geometric phase gate^8^ with its inherent robustness. Adiabatic protocols are also essential in quantum thermodynamics whether studying quantum fluctuations^9^ or the optimization of quantum thermal machines^10^,^11^,^12^,^13^. These applications are however limited by the requirement of slow driving that conflicts with the feebleness of quantum coherence when the system of interest is embedded in an environment.

According to the adiabatic theorem, a system prepared in a non-degenerate eigenstate will remain in the instantaneous eigenstate during its time evolution under the requirement of slow driving. By contrast, the breakdown of adiabatic dynamics under fast driving couples different energy modes and induces diabatic transitions. Diabatic excitations can however be tailored using shortcuts to adiabaticity (STA) to mimic adiabatic dynamics. Among the available techniques to engineer STA^14^, counterdiabatic driving (CD), relies on the use of an auxiliary control 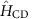 to explicitly suppress transitions between different energy eigenstates and enforce parallel transport^15^,^16^.

The transport can be realized by applying a time dependent force *f*(*t*) to a harmonic oscillator of mass *m* and frequency *ω*, which is described by


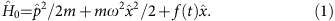


If we increase the force from zero to *f*(*t*) slowly, we can transport the ion over a distance *q*(*t*)=−*f*(*t*)/*mω*^2^. The excitations during the nonadiabatic transport can be seen in the instantaneous frame through the position-shift transformation 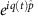, where we denote *ħ*≡1 throughout the manuscript. In the instantaneous frame, the time-dependent potential minimum is located at *x*=0 and the state is governed by the Hamiltonian 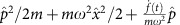, where a global phase term has been ignored. The first two terms describe the harmonic motion around the potential minimum. The last term is nonlocal in real space and induces diabatic transitions, vanishing only in the adiabatic limit. The CD suppresses these non-adiabatic transition without slowing down the dynamics by adding the auxiliary term^14^,^17^


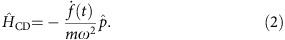


Because 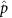 is invariant under the position-shift transformation, diabatic transitions are completely suppressed in the instantaneous reference frame under arbitrarily fast transport.

Here we experimentally realize the CD protocol for the nonadiabatic control of a single ^171^Yb^+^ ion (refs 18, 19) as it is transported in phase space. We use a pair of Raman beams to apply the force on the ion and achieve a precise and flexible control of the quantum evolution that allows us to unveil the superior performance of STA based on CD over alternative schemes. Our experiment provides a faithful realization of various STA protocols and is therefore complementary to previous studies on ion transport with time dependent electric fields^20^,^21^,^22^.

## Results

### Physical model and quantum control

**Figure 1:**
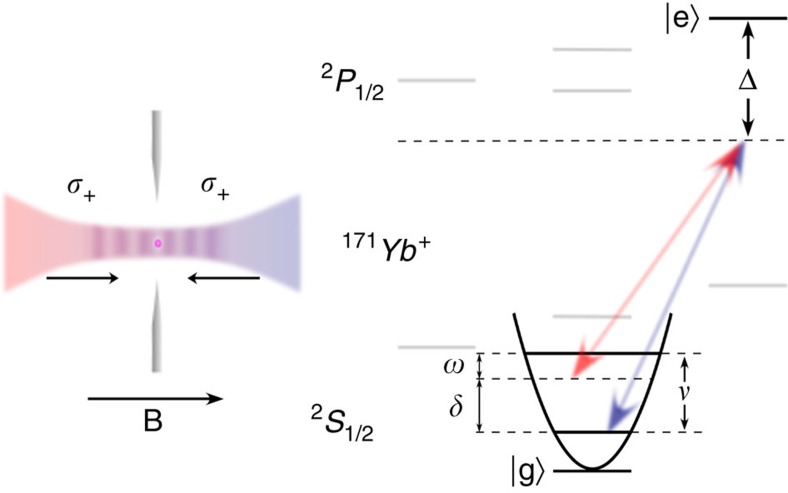
The scheme of the dragged harmonic oscillator model realized with a trapped ^171^Yb^+^. A pair of *σ*^+^ -polarized Raman laser beams with beat note *δ* counter propagate along the direction of the transversal motion and the magnetic field **B**. They are far (Δ≈2*π* × 14 THz) detuned from the exited state |*e*〉, which thus can be adiabatically eliminated. The moving standing wave formed by the lasers shakes the ion with the frequency *δ*, which is smaller than the transversal trap frequency *ν*=2*π* × 3.1 MHz by *ω*=2*π* × 20 kHz. In the rotational framework about the beat note frequency, the ion is dragged by the laser-induced force with an equivalent trap frequency *ω*. By varying the intensity and the phase of the Raman beams, we can control the direction and strength of the displacement of the ion in the phase space (energy levels are not to scale).

In the interaction picture with respect to the harmonic oscillation, the force induced by the lasers as configured in Fig. 1 is described by


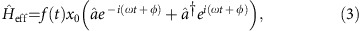


where 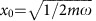, *f*(*t*)=Ω(*t*)Δ*k*/2, Ω(*t*) is proportional to the intensity of both Raman beams, Δ*k* is the projection of the wave-vectors difference of the Raman beams on the motional axis of the ion and *ϕ* is the phase difference between those two laser beams. Both laser beams are red detuned from the transition between the ground state 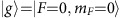 (^2^*S*_1/2_) and the excited state 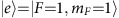 (^2^*P*_1/2_). Due to the large detuning Δ≈2*π* × 14 THz, the excited state 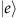 is adiabatically eliminated. The effective trap frequency *ω*=2*π* × 20 kHz in the interaction frame comes from the difference between the beat-note frequency of the laser beams *δ* and the real trap frequency *ν*=2*π* × 3.1 MHz. The effective mass is given by 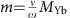 (*M*_Yb_: mass of ^171^Yb^+^). When the phase *ϕ*=0, the Hamiltonian (3) describes a dragged harmonic oscillator, with the dragging term 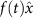. We can implement the CD term 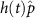, where 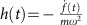, by setting *ϕ*=−*π*/2 (Supplementary Note 1).

### Counterdiabatic transport

**Figure 2:**
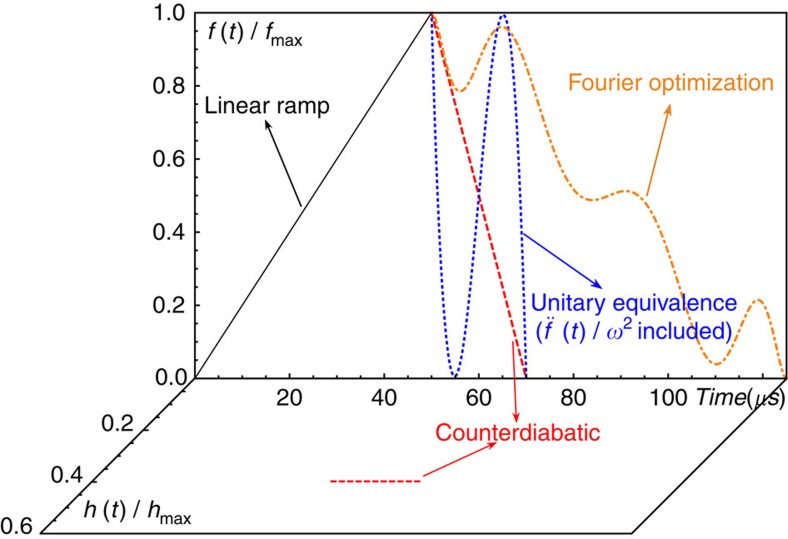
Time-dependent control fields for different STAs. To measure the phonon excitation after a shortcut, we first use a linear ramp within one period of the harmonic oscillation 2*π*/*ω*=50 μs. Then we apply different STA protocols to bring the ion back to its original location. The force *f*(*t*) is increased or decreased by changing the intensity of Raman laser beams with *ϕ*=0. The function *h*(*t*) represents the strength of the CD term proportional to the momentum, which is implemented by applying the laser beams with *ϕ*=−*π*/2 during the backward transport. The *f*_max_ and *h*_max_ are the maximum values allowed by the common maximum intensity of the laser beams. The smallest shortcut ratio is limited by the maximum laser intensity and we choose the value *s*=0.4 for the CD and UE transport and *s*=1.5 for the Fourier optimization scheme of degree *N*=3.

In the experiment, after Doppler and motional sideband cooling, 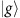 is prepared with 0.02±0.02 average phonon number. Because we cannot measure the phonon distribution in the interaction picture directly, the STA performance is probed with the quench echo method^23^ in which the ion is first transported adiabatically and then brought back to the initial location using the STA protocol. During the first adiabatic process, we linearly increase the force *f*(*t*) from 0 to *f*_max_=Ω_max_Δ*k*/2 within one period of the harmonic motion *T*_0_=2*π*/*ω*=50 *μ*s, where Ω_max_=2*π* × 378 kHz corresponds to the maximum value allowed by the laser. This linear ramp has been well studied in experiments^9^,^22^,^24^, and can be regarded as perfectly adiabatic. Following it, the force is linearly reduced from *f*_max_ to 0 within a duration of *sT*_0_, where *s* is defined as the shortcut ratio. The backward dynamics is assisted by turning on the laser to implement the CD term 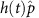 according to equation (2). The relation between the strength of the CD term and the shortcut ratio is given by *h*(*t*)≡*h*_max_/(2*πs*). Finally, we apply blue sideband transitions to measure the phonon distribution^18^. The time-dependent laser intensity profiles (waveforms) during the forward and backward transport stages are shown in Fig. 2. We vary *s* from 0.95 to 0.15 with a step of 0.1 and obtain the final average phonon number 0.016±0.018, which confirms that the CD protocol does not excite the motion after the transport for any duration (Supplementary Notes 2 and 3).

**Figure 3:**
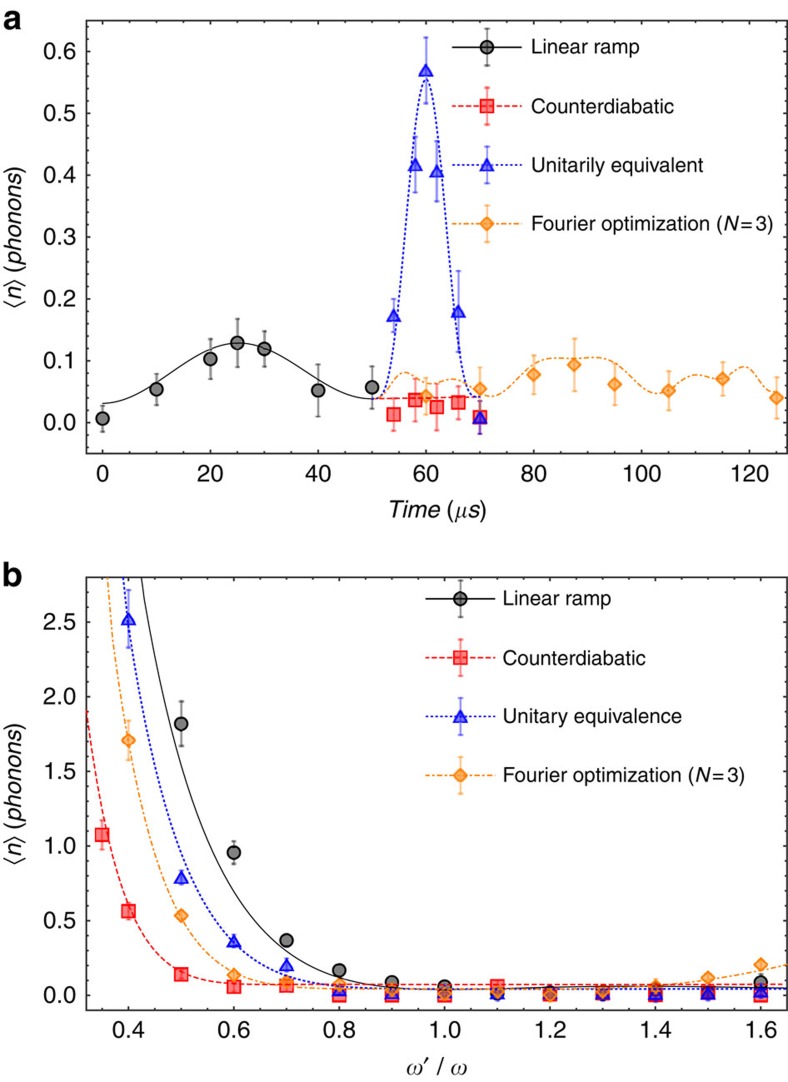
Phonon excitations in the instantaneous frame during different STA protocols and the robustness against trap frequency errors. (**a**) To measure nonadiabatic excitations during each STA, the shortcut waveform stops at a specific time and the system is transported back adiabatically to the initial position *q*=0 (see section B of Supplementary Information). This process brings phonon distributions back to the lab framework for the measurement. Note that only the CD realizes the adiabatic following. (**b**) To study the robustness of different STA protocols against the trap frequency drift, we change the trap frequency *ω* to *ω*′ during the shortcut transports, whose waveforms are still designed for the nominal trap frequency *ω*=2*π* × 20 kHz. Finally we measure the average values of excited phonons. For a fair comparison, we set *s*=1.5 for all three STA protocols. We also test the linear ramp method as reference. Finally the CD driving is found to be the most robust. The lines in both figures correspond to the numerical solution of the Lindblad master equation for the noise-average dynamics. The error bars represent the s.d. of 200 measurements.

We also measure phonon excitations in the instantaneous basis during the transport in order to certify that the dynamics is following the adiabatic ground state. During the forward linear ramp and the backward CD transport, we stop at different instants and add another CD transport with *s*=0.15 to adiabatically change back to the lab frame. As shown in Fig. 3a, we do not observe any significant excitation during the transport, which confirms that the CD is speeding up the adiabatic trajectory associated with 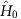 as in a ‘fast motion video'. We also measure the excitation in the lab framework (Supplementary Note 4).

Furthermore, the CD is shown to be robust against the trap frequency drift error. We design STA's waveforms with the nominal trap frequency *ω*=2*π* × 20 kHz. In the first linear adiabatic ramp, we keep the trap frequency *ω*, but change the effective trap frequency to *ω*′ during the STA transport. Then we measure the final average phonon excitation as a function of *ω*′/*ω*. The result in Fig. 3b shows that the CD is extremely robust against the drift of the trap frequency. This feature can be qualitatively explained by the results shown in Fig. 3a, where almost no excitation appears during the CD driving. Since the higher excited states are more fragile to errors, the protocol with the smaller excitations during the transport is surely more robust. The higher robustness also results from the lower amplitude of the required control field. In the experiment, for the shortcut ratio *s*=0.4, the CD protocol uses three times less intensity than the other protocols, which naturally reduces the amount of noise proportionally.

### Unitarily equivalent transport

The CD stands out among STA protocols for its robustness and the adiabatic following during the whole evolution. Yet, the realization of the auxiliary control 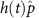 is hardly feasible with classical electrical fields. Many efforts have been devoted to identify alternative controls requiring only local potentials^14^,^17^,^25^,^26^,^27^. To this end, we resort to controls related to CD via its unitary equivalence (UE)^14^,^17^,^25^,^28^. The exact solution to time-dependent Schrödinger equation with Hamiltonian 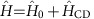 is given by the adiabatic approximation 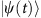 to the dynamics generated by 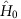. Under a momentum-shift transformation 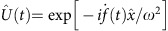, the time evolving state becomes 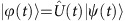, which is governed by the Hamiltonian,


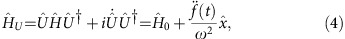


where a global phase term has been gauged away. The auxiliary control in the driving Hamiltonian 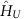 can be realized with a local potential. As long as 
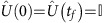
, the state 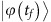
reproduces exactly the desired target state 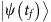 on completion of the STA protocol. This suggests a route to design the UE transport waveform. The boundary conditions *f*(0)=0 and *f*(*t*_*f*_)=*f*_max_ define the transport problem. Vanishing first-order derivatives 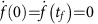 guarantee that 
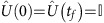
. Considering that generally we do not suddenly turn on or off control fields, we further impose second-order boundary conditions 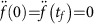. These constraints are satisfied by a polynomial waveform *f*(*t*)=10(*t*/*t*_*f*_)^2^−15(*t*/*t*_*f*_)^3^+6(*t*/*t*_*f*_)^4^ (refs 17, 29). In the experiment, we apply the UE transport in the backward process, as shown in Fig. 2.

For the UE transport, we measure the final average phonon number 0.026±0.019 for various shortcut ratios *s* from 1 to 0.4 with a step of 0.1. As shown in Fig. 3a, we also examine the process of the UE transport in the instantaneous basis and in the lab framework (Supplementary Note 4). We observe large excitations in the process, which shows the UE protocol does not follow the adiabatic evolution, but succeeds in preparing the adiabatic target state at the final stage. As shown in Fig. 3b, the robustness against the drift of the trap frequency is below that of the CD transport. Note that the *f*(*t*) used is not the only solution. Simulation results (Supplementary Note 5) show that the waveform will be more sensitive to the trap frequency error, when higher order boundary conditions are considered. The first-order polynomial waveform can also be used to mimnimize the DC Stark shift during the transport with the electric fields^30^.

### Fourier optimization transport

Finally, we implement the Fourier optimization scheme as proposed in^27^. When the applied force *f*(*t*) for transport satisfies the conditions *f*(0)=0 and 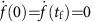, the final excitation energy can be expressed as the Fourier transform of the acceleration of the force at the trap frequency. In principle, this method allows us to find a driving *f*(*t*) that simultaneously minimizes the final excitation energy for an ensemble of *N* different trap frequencies. When they are equalized, the final excitation is set by (*ω*′^2^−*ω*^2^)^*N*^, which enhances the robustness with *N*. The cost of the enhanced robustness is the increase of the amplitude of the control field with the order *N*. In our experiment, we choose *N*=3 that results in a oscillatory waveform, shown in Fig. 2. The required amplitude of the control field greatly surpasses *f*_max_ for a small shortcut ratio, thus we only test the scheme for *s*=1.5. The excitation in the instantaneous base and its robustness are shown in Fig. 3a,b, respectively.

## Discussion

We have provided a realization of shortcuts to adiabaticity based on counterdiabatic driving in a continuous variable system. By demonstrating the robust adiabatic following, we have shown that the resulting time-evolution follows a ‘fast-motion video' of the adiabatic dynamics. This protocol is also known to be the optimal solution of the quantum brachistochrone problem^31^. We have further realized two competing STA protocols for the transport problem: local UE driving and Fourier optimization methods. In the UE scheme, while the auxiliary control field takes the form of a time-dependent linear potential, its amplitude scales as 
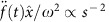
 surpassing the value required for counterdiabatic driving, 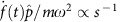. We note that by further modulating the trap frequency during transport, these shortcuts can still be accelerated within a maximum control field with the ‘rapid scan method', that has been realized for a two level system^28^. The total duration can then be reduced to half for the tested UE protocol reported here. As for the Fourier optimization scheme, its robustness is reduced even with respect to the UE scheme for a given amplitude of the control field, but could be increased with the order *N* and a higher amplitude of the control field (Supplementary Note 6).

In our experiment, we demonstrate that the challenging non-local CD term can be generated in the interaction frame. Therefore, our results will be directly influential and beneficial to the other experimental works that require adiabatic evolution in short time and are performed in the interaction picture including quantum thermodynamics, quantum simulation and quantum computation. The transport of a harmonic oscillator can be a test bath for quantum thermodynamics^9^ or as part of a quantum engine^11^,^12^,^13^, for which the CD protocol can be used to boost the performance. For many quantum-simulation experiments, adiabatic evolution is essential to prepare a complex ground state of non-trivial Hamiltonian from a simple Hamiltonian whether or not in the interaction frame. The non-trivial ground state of a bosonic Hamiltonian or spin-boson Hamiltonian could be implemented via the CD protocol, overcoming the limitation imposed by the coherence time of the system. The CD protocol can also speed up routines in holonomic quantum computation^7^,^8^,^32^,^33^, and enable the implementation of topological quantum computation with non-Abelian braiding operations^34^ that need not be adiabatic.

## Methods

### The dragged harmonic oscillator model

As mentioned in the main text, the Hamiltonian of the dragged harmonic oscillator in the interaction picture about the harmonic motion is equation (3). Here we apply a pair of Raman beams to the ion with a beatnote, which is red detuned to the real trap frequency *ν* with the nominal trap frequency *ω* to simulate this Hamiltonian. We can find the detail of the laser ion interaction in the section A of the Supplementary Information. And the interaction Hamiltonian is 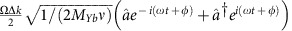, which equals the equation (3) when *f*(*t*)=Ω(*t*)Δ*k*/2 and 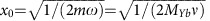, where the effective mass 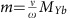.

### Dynamics in the instantaneous basis

To study the STA dynamics we measure phonon excitations in the instantaneous basis during the transport, and use a short CD protocol to change to the lab frame, where the measurements can be made. To choose the protocol for the frame change, we measure the fidelity of different STA with various shortcut ratios (Supplementary Note 2) and find that the CD transport with the smallest shortcut ratio *s*=0.15 is optimal. In addition to its robustness against the trap frequency error, its shortest duration protects the motion of the ion from the heating effect.

### Data availability

Raw data for any of the results reported in the text are available from the authors on request.

## Additional information

**How to cite this article:** An, S. *et al*. Shortcuts to adiabaticity by counterdiabatic driving for trapped-ion displacement in phase space. *Nat. Commun.* **7,** 12999 doi: 10.1038/ncomms12999 (2016).

## Supplementary Material

Supplementary Information Supplementary Figures 1-7, Supplementary Notes 1-6 and Supplementary References.

Peer Review File
