# Supplementary material for: Shortcuts to adiabaticity by counterdiabatic driving for trapped-ion displacement in phase space
Source: Nat Commun. 2016 Sep 27;7:12999. doi: 10.1038/ncomms12999 (PMC5052658; doi:10.1038/ncomms12999)
Supplement: Supplementary Information — Supplementary Figures 1-7, Supplementary Notes 1-6 and Supplementary References. [file ncomms12999-s1.pdf]

Reviewer #1 (Remarks to the Author):

This paper demonstrates the use of shortcuts to adiabaticity (STA) to to perform non-adiabatic control of an engineered trapped ion oscillator in an interaction picture which is created in the experiment. The experiment is set up to produce an artificial low effective trap frequency which allows the non-adiabatic regime to be achieved with low demands on the control fields. This allows the investigation and comparison of a number of STA schemes, and the authors perform an in-depth investigation.

STA have been explored extensively theoretically. In this paper a specific scheme is demonstrated in an experimental setting, which is the translation of a single trapped ion in an engineered interaction-picture phase space. As a result of the excellent level of control which is achievable with trapped ions, the experimental results reproduce the theoretical predictions well, including demonstrating the robustness of the scheme to changes in the effective trap frequency, which is one important factor in control. The authors also make considerations which are relevant to transport of ions using electric field in traps, demonstrating a "Unitarily equivalent" control which performs a similar role to the CD scheme.

While the area of STA is interesting for its potential in quantum state control, I am not convinced that this article has a suitable impact for publication in Nature Communications. The reason for this is that the experimental implementation is for a contrived situation in which care has been taken to reproduce exactly the conditions where STA has an advantage (even to the extent of moving to an interaction picture in which an effective trap frequency arises which is low compared to the classical laser control speeds). No new experimental capability is demonstrated that is useful outside this demonstration, and the demonstration does not access any parameter space which is beyond previous work. For this reason, while the application in trapped ions may draw more attention to STA within this community, it isn't likely to provoke any interest beyond that which was already present due to the previous theoretical proposals. I would be more supportive if there were obvious benefits directly transferrable from the experimental work for quantum simulation, computation, or even transport of ions, which are practical tasks, or if new states were accessed that were unavailable previously. However this is not the case in the current manuscript.

Barring the above comments on the nature of the advance, the paper is generally well written, and error bars etc. are treated appropriately. I have a few points of improvement which are given below.

One point on presentation.

Figure 2. This has a strange format in which one half of the plot (which corresponds to the CD term) being laid at a strange and difficult to read angle. I cannot see why this is done, since it would be much easier to read if the two plots were laid one above the other. The plot for h(t) also seems to only feature one horizontal line. Is this really worth a plot?

A couple of text notes follow.

Page 1, column 2. The phrase "Note that we ignore a global phase term" is followed by "The CD suppresses it". I would change the latter to "The CD term suppresses these non-adiabatic transition without slowing down the dynamics through the addition of an auxilliary term..."

Page 3, last sentence. Would be better written as "The first-order polynomial waveform can also be used to mimnimize the DC Stark shift during..." I think the authors should note that the DC Stark shift is only likely to be a problem in the most extreme of circumstances.

Reviewer #2 (Remarks to the Author):

In the manuscript the experimental implementation of counterdiabatic driving in a continuous variable system is reported, and applied to the parallel transport of an ion confined in a harmonic trap. The result is sound and of interest and in my opinion deserves publication on Nature Communication. However in my opinion some more effort is needed to discuss the robustness of the protocol. I suggest that the paper may be reconsidered after some revision in this direction.

Although obtained by a sophisticated advanced control procedure, the protocol by itself is very simple, amounting to the transfer of the population between the ground states of a displaced ocillator. Therefore robustness is a major issue and it would be desirable a detailed analysis. In particular I would suggest what follows.
(1) Robustness with respect to variations of the natural frequency of the trap has been studied. A qualitative analysis of the results of fig. 3b should appear in the main text. I assume lines in fig 3 are results of simulations, ad this should be stated.
(2) Are there any other sources of parametric errors ? For instance what could affect the position of the minimum of the trap and how to estimate or measure the error ?

Reviewer #3 (Remarks to the Author):

The main result of this work is the first experimental implementation of counterdiabatic driving in a continuous variable system. More precisely, the authors realized a shortcut to the adiabaticity transport of a trapped ion.
This particular system has allowed for the first time to engineer a counterdiabatic driving Hamiltonian to supress transitions between different eigenstates and enforce parallel transport. Thus this work is novel and very interesting for the whole optimal control community in general, and for communities working on ion manipulation, such as the quantum information community.

The authors compared the counterdiabatic transportwith the unitary equivalent transport and the Fourier optimization transport, showing the robustness and the superiority of the counteradiabatic transport with respect to the other technics.

The paper is very clear and the results are very convincing and robust.

I support, without any hesitation, the publication of this manuscript as Nature communication. No corrections are requested.

____

Summary of changes and reply to Referee's report

Reviewer #1:

Comment:

This paper demonstrates the use of shortcuts to adiabaticity (STA) to perform non-adiabatic control of engineered trapped ion oscillator in an interaction picture which is created in the experiment. The experiment is set up to produce an artificial low effective trap frequency which allows the regime to be achieved with low demands on the control fields. This allows the investigation and comparison of a number of STA schemes, and the authors perform an in-depth investigation.

Response:

We appreciate the summary by the reviewer on our report of the first experimental demonstration of counterdiabatic driving in a continuous variable system. Indeed, the supremacy of this protocol over alternative shortcuts to adiabaticity, that we demonstrate experimentally, results from the low demands on the control fields. Common expectation is that the auxiliary term of the CD driving tends to be non-local and highly complicated, and as such CD has remained a theoretical construct. This understanding has recently been challenged by theoretical advances suggesting its feasible implementation in relevant scenarios in quantum science and technology, ranging from the driving of quantum fluids in ultracold atom physics to the adiabatic crossing of a quantum phase transition or the design of quantum logic gates. In our experiment, we manage to fully utilize the controllability of our setup to fulfill all the requirements for a faithful realization of CD protocol and report this primary test.

Comment:

While the area of STA is interesting for its potential in quantum state control, I am not convinced that this article has a suitable impact for publication in Nature Communications.
The reason for this is that the experimental implementation is for a contrived situation in which care has been taken to reproduce exactly the conditions where STA has an advantage (even to the extent of moving to an interaction picture in which an effective trap frequency arises which is low compared to the classical laser control speeds).

Response:

The main merit of our demonstration is that it is the first experimental test of the counterdiabatic (CD) techniques including continuous variables systems. As shown in our experiments, the CD protocol occupies a unique status among other STA protocols. There has been increasing number of studies of the CD protocol for the transport of a harmonic oscillator, which is a natural choice for the primary test of the theory of CD as shown in many references quoted in our manuscript. Up to now, however, there is no experimental test for the CD protocol even for a contrived situation. Our experimental demonstration contains the first explicit CD protocol and manifests its main fundamental features: a) the adiabatic following of the ground state of the instantaneous system Hamiltonian all the time during the process, no matter how fast the

transport is, and b) its superior robustness over alternative STA schemes. We believe that our demonstration will be the guiding reference for other realizations.

Comment:

No new experimental capability is demonstrated that is useful outside this demonstration, and the demonstration does not access any parameter space which is beyond previous work. For this reason, while the application in trapped ions may draw more attention to STA within this community, it isn't likely to provoke any interest beyond that which was already present due to the previous theoretical proposals.

I would be more supportive if there were obvious benefits directly transferrable from the experimental work for quantum simulation, computation, or even transport of ions, which are practical tasks, or if new states were accessed that were unavailable previously. However this is not the case in the current manuscript.

Response:

As we mentioned above, the CD protocol achieves an outstanding performance among alternative schemes, which makes it of primary interest for experimental advances in quantum technology. However, these advantages come with the challenge to create the non-trivial counterdiabatic term that has hindered the experimental realization before us. In our experiment, we move from the lab frame to an interaction frame, where we are able to generate the non-local counterdiabatic term in a controllable way. In addition, we succeed in doing so in a continuous variable system, despite its higher complexity, e.g., over few level systems.

Therefore, we believe our demonstration would influence many other situations that require adiabatic evolution in a short time. In our previous demonstration of Ref. [9], for example, it is necessary to transfer the energy eigenstates from the interaction frame to those in lab frame, where the duration of the transfer would be significantly reduced through the application of the CD protocol. Similarly, the CD protocol can be used to test universal principles in quantum thermodynamics or the implementation of quantum engine shown in Refs. [11,12,13] in the revised manuscript. Moreover, our CD protocol is applicable and beneficial to many types of quantum simulations, where adiabatic evaluation is essential to prepare a complex ground state of non-trivial Hamiltonian from a simple Hamiltonian, which could be in the interaction picture. A squeezed state, Schrodinger cat state or more complex states can be prepared through adiabatic process assisted by the CD protocols. For the quantum computation, the CD protocol would enhance the performance of holonomic quantum computation [7,8,33,34], which requires the adiabatic evolution or enable us to implement topological quantum computation with the adiabatic non-Abelian braiding operations [PRB 91, 201102(R) (2015), which is #35 in the revised manuscript].

In case that the implementation of the CD term is not all available, the unitarily equivalent (UE) to the CD protocol would be the preferable choice as a STA protocol, which is discussed in our manuscript. In the UE protocol, the implementation is less demanding than the CD protocol at

the expense of a lower robustness, as we have demonstrated. The UE scheme is directly applicable and beneficial to the transport of ions by electric field reported in Refs. [20,21], where the term of non-local momentum operator is not required for the implementation.

We believe that these examples of CD and UE protocols clearly show obvious and immediate benefits for quantum thermodynamics, quantum simulation, quantum computation and ion transport.

To address these points, we include additional paragraph at the conclusion section.

"In our experiment, we demonstrate that the challenging non-local CD term can be generated in the interaction frame. Therefore, our results will be directly influential and beneficial to the other experimental works that require adiabatic evolution in short time and are performed in the interaction picture including quantum thermodynamics, quantum simulation, and quantum computation. The transport of a harmonic oscillator can be a test bath for quantum thermodynamics [9] or as part of a quantum engine [11-13], for which the CD protocol can be used to boost the performance. For many quantum-simulation experiments, adiabatic evolution is essential to prepare a complex ground state of non-trivial Hamiltonian from a simple Hamiltonian whether or not in the interaction frame. The non-trivial ground state of a bosonic Hamiltonian or spin-boson Hamiltonian could be implemented via the CD protocol, overcoming the limitation imposed by the coherence time of the system. The CD protocol can also speed up routines in holonomic quantum computation [7,8,33,34], and enable the implementation of topological quantum computation with non-Abelian braiding operations [35] that need not be adiabatic. "

Comment:
Barring the above comments on the nature of the advance, the paper is generally well written, and error bars etc. are treated appropriately. I have a few points of improvement which are given below.
One point on presentation.
Figure 2. This has a strange format in which one half of the plot (which corresponds to the CD term) being laid at a strange and difficult to read angle. I cannot see why this is done, since it would be much easier to read if the two plots were laid one above the other. The plot for h(t) also seems to only feature one horizontal line. Is this really worth a plot?

Response:
We thank the referee for his/her suggestions to improve the manuscript. The h(t) is one part of the CD control. It should be plotted in order not to mislead the reader as it constitutes a different control from a linear potential. At first we plot the h(t) with all the other f(t) in the same plan. But we find it looks cluttered and cannot clearly show the orthogonal relationship between the force term and the CD term. But the fig. 2 in current way can resolve these problems.

Comment:
A couple of text notes follow.

Page 1, column 2. The phrase "Note that we ignore a global phase term" is followed by "The CD suppresses it". I would change the latter to "The CD term suppresses these non-adiabatic transition without slowing down the dynamics through the addition of an auxiliary term..."

Response:
We appreciate the reviewer's careful reading and already corrected this sentence.

Comment:
Page 3, last sentence. Would be better written as "The first-order polynomial waveform can also be used to minimize the DC Stark shift during..." I think the authors should note that the DC Stark shift is only likely to be a problem in the most extreme of circumstances.

Response:
We thank the reviewer for the comment and we have already changed the sentence following the suggestion. We state this feature of the first-order polynomial waveform because we find the previous work [29] found the same transport waveform with the optimal control method instead of the STA theory. This coincidence is inspiring and gives a practically good property, which may attract people to use it when the ion is transported with the DC electric field.

Reviewer #2:

Comment:
In the manuscript the experimental implementation of counterdiabatic driving in a continuous variable system is reported, and applied to the parallel transport of an ion confined in a harmonic trap. The result is sound and of interest and in my opinion deserves publication on Nature Communication.

Response:
We appreciate the reviewer's positive opinion about our work and her/his support for publication in Nature Communication.

Comment:
However in my opinion some more effort is needed to discuss the robustness of the protocol. I suggest that the paper may be reconsidered after some revision in this direction.
Although obtained by a sophisticated advanced control procedure, the protocol by itself is very simple, amounting to the transfer of the population between the ground states of a displaced oscillator. Therefore robustness is a major issue and it would be desirable a detailed analysis.

Response:
We gave found the reviewer's comments below very helpful to improve our manuscript. We have followed the suggestions by the referee and include an analysis of the robustness of the protocols, as discussed below.

Comment:

In particular I would suggest what follows.
(1) Robustness with respect to variations of the natural frequency of the trap has been studied. A qualitative analysis of the results of fig. 3b should appear in the main text. I assume lines in fig 3 are results of simulations, ad this should be stated.

Response:
We add a qualitative analysis of the results of fig. 3 at the last paragraph of the section **Counterdiabatic transport**: "This feature can be qualitatively explained by the results shown in Fig. 3(a), where almost no excitation appears during the CD driving. Since the higher excited states are more fragile to errors, the protocol with the smaller excitations during the transport is surely more robust. The higher robustness also results from the lower amplitude of the required control field. In the experiment, for the shortcut ratio s=0.4, the CD protocol uses three times less intensity than the other protocols, which naturally reduces the amount of noise proportionally."

We now clearly state the meanings of the plotted curves in fig. 3 in the caption to which we have added the sentence: "The lines in both figures correspond to the numerical solution of the Lindblad master equation for the noise-average dynamics.''

Comment:
(2) Are there any other sources of parametric errors? For instance what could affect the position of the minimum of the trap and how to estimate or measure the error?

Response:
The errors in the experiment can be classified into two categories, where one is the operational error and the other is the environmental error. For the operational error, the fluctuations of laser intensity and frequency are representative sources of errors, which also affect the position of the minimum in the phase space. Since we always align the center of the ion and the laser beam in the beginning of the laser transport, the actual position of the ion is not important in our experimental study. The laser frequency drift is effectively equivalent to that of trap-frequency, which we carefully study and describe in the manuscript. For the intensity of the laser beam, it is stabilized within the fluctuation of 1-2%. Since our protocol ends with zero laser intensity, the effect of the intensity fluctuations should be minimal, which is shown in low average phonon in the final stage.
For the environment error, the most important error is the heating effect of the motion, which is believed to be caused by the patch potential of the electric field and can be regarded as a fast and random position shift of the ion. People can improve the trap quality to reduce this error. We'd like to emphasize that the STA is a good way out of this problem when the adiabatic process is required. The shorter duration will give the results less affected by the heating effect. And in the same duration, the final average phonon of the CD protocol is generally lower than that of the UE, which takes longer duration than CD.

Reviewer #3:

Comment:

The main result of this work is the first experimental implementation of counterdiabatic driving in a continuous variable system. More precisely, the authors realized a shortcut to the adiabaticity transport of a trapped ion.
This particular system has allowed for the first time to engineer a counterdiabatic driving Hamiltonian to suppress transitions between different eigenstates and enforce parallel transport.
Thus this work is novel and very interesting for the whole optimal control community in general, and for communities working on ion manipulation, such as the quantum information community.
The authors compared the counterdiabatic transport with the unitary equivalent transport and the Fourier optimization transport, showing the robustness and the superiority of the counterdiabatic transport with respect to the other technics.
The paper is very clear and the results are very convincing and robust.
I support, without any hesitation, the publication of this manuscript as Nature communication. No corrections are requested.

Response:

We thank the referee for his/her pertinent summary of our work and the positive comment about our work, which she/he find clear and reporting convincing results. We also appreciate her/his recommendation for the publication of our work without hesitation in Nature Communications.

Reviewer #1 (Remarks to the Author):

This is the second time that I have seen this paper. In their response to my original criticism, the authors argue for the general applicability of Counteradiabatic Driving, and their modification to the paper reflect this. I have no problem with the general applicability of this method, which I think is useful, and this is reflected in the wide theoretical literature. My criticism relates to the significance of this particular experimental demonstration.

How does the current experimental demonstration add to the literature? This could be either through perspectives on the limitation of the theory, by adding an experimental capability to our current set which is advantageous for further experiments, or by showing an ability to explore quantum physics beyond other experiments. The experiment has been explicitly set up so that it can replicate the theory as closely as possible, to the extent of working in a particular rotating frame with an artificially low effective trap frequency. Given that trapped ion work is able to accurately reproduce theory if performed correctly, it is natural that the protocol is thus seen to work. For the experimental advance the experiment is so contrived as to reduce/remove the applicability. The author's response completely fails to provide a perspective on how their experiment would be extended to access any of the interesting claimed physics mentioned for CD. In this sense, they highlight the limitations of the paper rather than making it stronger. I cannot therefore recommend publication in a high profile journal such as Nature Communications.

It is also worth noting that the title misleadingly claims an effect on trapped ion transport (an ingredient expected to be important for trapped-ion quantum information processing). In the experiment the ion does not get transported at all in the traditional sense (transport usually implies that the centre of mass of the ion averaged over many oscillation cycles is non-zero), but is displaced in the rotating phase space. This is very different from real experiments on ion transport where people made significant efforts to be able to move ions trapped in potential wells in a trap array both diabatically and adiabatically. To put this in perspective, to apply CD methods in real transport the control would need to be performed on timescales more than 100 times faster than the current work for typical trap frequencies (this would be a genuinely challenging experimental task). This makes the title quite misleading and this should certainly be changed, even if Nature Communications decides to publish this paper. Maybe using "for phase-space displacements of trapped ions" instead of "in Trapped-Ion Transport" would be a better choice.

Reviewer #2 (Remarks to the Author):

Changes in the revised version of the maniscript are appropriate. I expected some more discussion on robustness. In particular my question (2) on other sources of parametric errors was intended to be answered in the manuscript. However I understand that readability could suffer from it.
I think that it is mandatory to mention other sources of parametric errors in the text, which can be discussed in the supplemental material.
When these changes are made, the manuscript can be published on Nature Communications with no further review on my side.

Summary of changes and reply to Referee's report

Reviewer #1:

Comment:

This is the second time that I have seen this paper. In their response to my original criticism, the authors argue for the general applicability of Counteradiabatic Driving, and their modification to the paper reflect this. I have no problem with the general applicability of this method, which I think is useful, and this is reflected in the wide theoretical literature. My criticism relates to the significance of this particular experimental demonstration.
How does the current experimental demonstration add to the literature? This could be either through perspectives on the limitation of the theory, by adding an experimental capability to our current set which is advantageous for further experiments, or by showing an ability to explore quantum physics beyond other experiments. The experiment has been explicitly set up so that it can replicate the theory as closely as possible, to the extent of working in a particular rotating frame with an artificially low effective trap frequency. Given that trapped ion work is able to accurately reproduce theory if performed correctly, it is natural that the protocol is thus seen to work. For the experimental advance the experiment is so contrived as to reduce/remove the applicability. The author's response completely fails to provide a perspective on how their experiment would be extended to access any of the interesting claimed physics mentioned for CD. In this sense, they highlight the limitations of the paper rather than making it stronger. I cannot therefore recommend publication in a high profile journal such as Nature Communications.

Response:

The reviewer's main concern is the "*significance of this particular experimental demonstration.*"
We should say that this work is the *first* demonstration that shows the CD really works and it works better than other control protocols. It is important to the field of STA and quantum control. This is particularly the case as counterdiabatic driving (or transitionless driving or super-adiabatic driving) is considered as the origin of the whole field of STA, ``the mother technique" to design STA. The previous two experiments (Bason, M. G. et al. Nature Physics 8, 147–152 (2012) and Zhang, J. et al. Phys. Rev. Lett. 110, 240501 (2013)) only considered the UE for the two-level systems. The main limitation of the application of the CD is the complicated form of the auxiliary Hamiltonian. For example, in a chain of N spins the CD term will contain the product of N different \sigma_(-,+ or x,y) operator of each spin in the Ising or XY model. In other cases, CD turns out to be accessible. This is the case in two (or three) level systems and the harmonic oscillator but as well in remarkably complex instances such as strongly correlated quantum fluids. Here we choose the harmonic oscillator to realize the first demonstration of CD, a complementary effort to the previous two experiments. Before we go to the more complicated many (or few) body systems, which should be the real task the reviewer said before, we should firstly see the CD works for the relatively simple and universal system. At the same time, some common

features of different STA have been revealed in this work. For example, the same way as we get the UE form from the CD has been reported very recently by theorists (A. Baksic et al. PRL 116, 230503 (2016)). Considering all these publications, we believe our work deserves the publication in Nature Communication.

Comment:

It is also worth noting that the title misleadingly claims an effect on trapped ion transport (an ingredient expected to be important for trapped-ion quantum information processing). In the experiment the ion does not get transported at all in the traditional sense (transport usually implies that the centre of mass of the ion averaged over many oscillation cycles is non-zero), but is displaced in the rotating phase space. This is very different from real experiments on ion transport where people made significant efforts to be able to move ions trapped in potential wells in a trap array both diabatically and adiabatically. To put this in perspective, to apply CD methods in real transport the control would need to be performed on timescales more than 100 times faster than the current work for typical trap frequencies (this would be a genuinely challenging experimental task). This makes the title quite misleading and this should certainly be changed, even if Nature Communications decides to publish this paper. Maybe using "for phase-space displacements of trapped ions" instead of "in Trapped-Ion Transport" would be a better choice.

Response:

We are sorry for the feeling of misleading and have changed the title and the abstract accordingly, so that it reads

"Shortcuts to Adiabaticity by Counterdiabatic Driving for Trapped-ion Transport in Phase Space"

Reviewer #2:

Comment:

Changes in the revised version of the manuscript are appropriate. I expected some more discussion on robustness. In particular my question (2) on other sources of parametric errors was intended to be answered in the manuscript. However I understand that readability could suffer from it.
I think that it is mandatory to mention other sources of parametric errors in the text, which can be discussed in the supplemental material.
When these changes are made, the manuscript can be published on Nature Communications with no further review on my side.

Response:

The other sources of parametric errors could be the error in the applied force. There are two

typical situations for this error. If the laser power drift, the force or the momentum term will drift also. But during one experiment run, the laser power can still be regarded as fixed. Such unitary control error will not increase the final excitation, which is different from the trap frequency drift. This force drift error can be regarded as the transport distance has been rescaled. As a result, the wrong CD term is still the right CD term for the wrong force. So we did not test for this error. The other situation is the heating effect caused by the fast, random and small force from the environment as we have mentioned in the previous reply. The STA may be the best way out for beating the resulting decoherence. The heating effect for different phonon states has been studied and the experiment shows the higher phonon states are more fragile. From the Supplementary Figure 4(b) and 4(d), we know in the lab framework, the CD will excite less phonons than the UE. That means the CD should be more robust. But the heating effect is so small in such a short STA duration that we almost cannot clearly see the difference. Maybe the small difference in the final excitation in the Supplementary Figure 2 can be a qualitative evidence. For our system, the heating rate is almost not tunable without changing other parameters. So we did not test the robustness of different STAs for different heating rates.

Following the suggestion by the referee, we have added a more detailed discussion in the Supplementary Note 4, that now reads:
From Supplementary Figure 4, we also find the UE will excite more phonon than the CD in the lab framework. Because the higher phonon states are more fragile [1], we expect the CD will be more robust against the heating effect. This can be the reason for the tiny difference between the final excitations showed in Supplementary Figure 2.
