# Supplementary material for: Shortcuts to adiabaticity by counterdiabatic driving for trapped-ion displacement in phase space
Source: Nat Commun. 2016 Sep 27;7:12999. doi: 10.1038/ncomms12999 (PMC5052658; doi:10.1038/ncomms12999)
Supplement: Peer Review File [file ncomms12999-s2.pdf]

SUPPLEMENTARY FIGURES

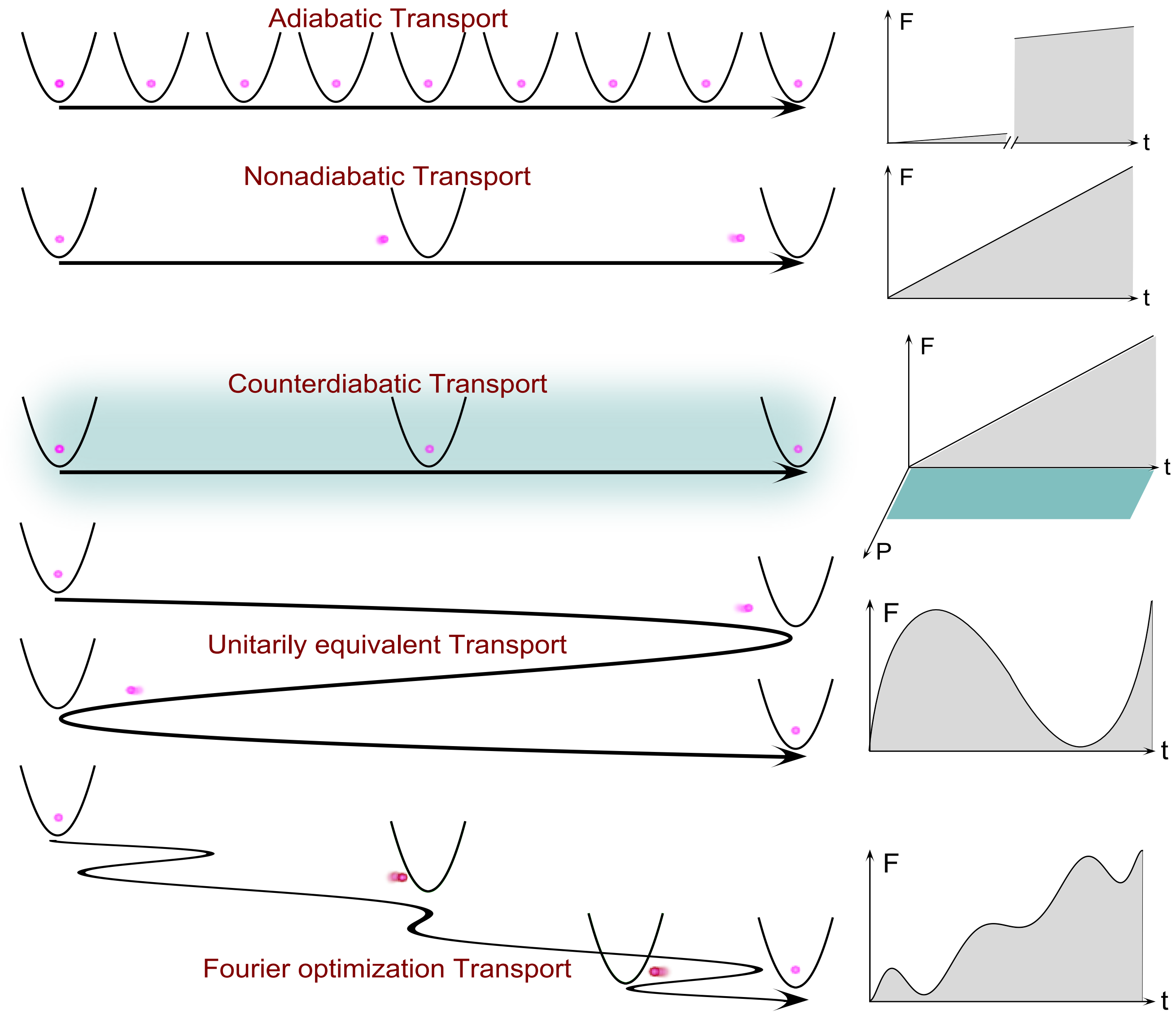

Supplementary Figure 1: Illustration of the different transport protocols experimentally studied. The panels in the right hand side display the time-dependent force applied in the different driving schemes.

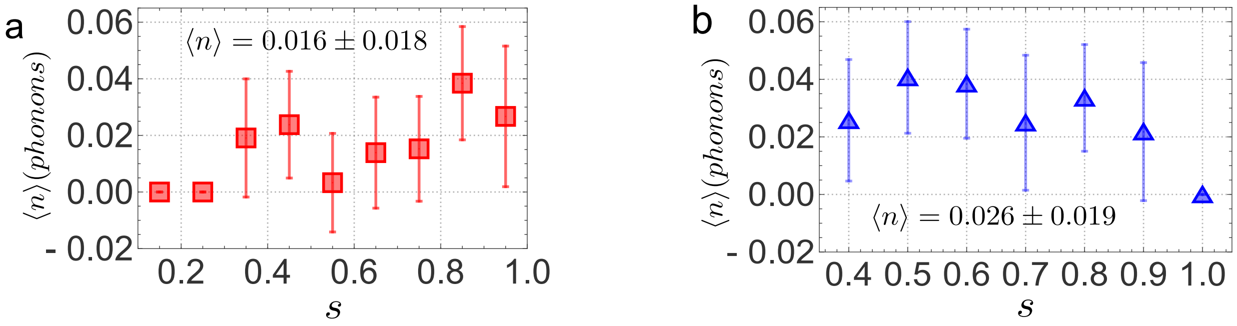

Supplementary Figure 2: **Performance of STAs depending on the shortcut ratio.** (a) We apply the CD transport with different shortcut ratios to bring the ion back after the first linear ramp. The final phonon excitations are recorded against different shortcut ratios. We do not observe any degrade of the performance of the CD protocol for the minimum $s = 0.15(\approx \frac{1}{2\pi})$ allowed by the maximum laser intensity. (b) We apply the UE transport with different shortcut ratios to bring the ion back after the first linear ramp. The final phonon excitations are recorded against different shortcut ratios. The curves are numerical simulated results and the points are the experiment results. The performance of the UE is good even for the minimum $s = 0.4$ allowed by the maximum laser intensity. Note that the smallest shortcut ratio s for the UE is larger than that of the CD protocol and the final excitations are larger than those of the CD protocols, which indicates the CD protocol is more robust that the UE protocol. The error bars represent the standard deviation of 200 measurements of the average phonon excitation.

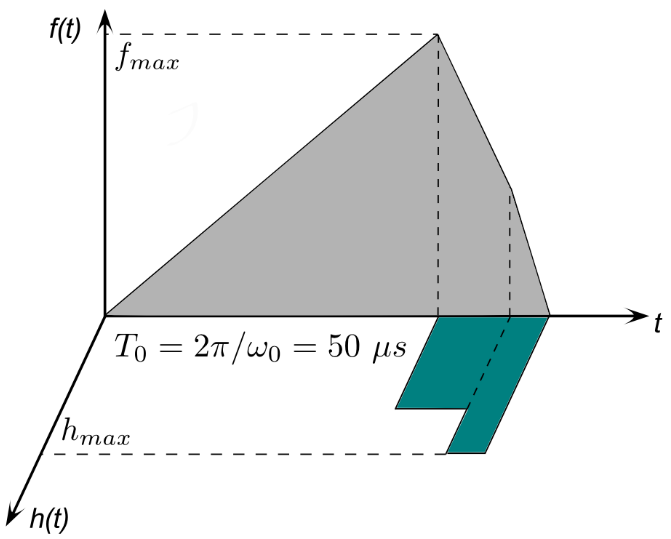

Supplementary Figure 3: **The measurement in the instantaneous basis.** In the forward process, we linearly increase the force from zero to f_{max}. Then we apply the CD protocol to bring the ion back to the original place. In order to measure the phonon excitation in the instantaneous frame, we apply the additional CD protocol in the end. The final CD transport has the shortcut ratio $s = 0.15$. We also apply the same CD protocol to study the dynamics of UE protocol and the Fourier optimization scheme in the instantaneous basis.

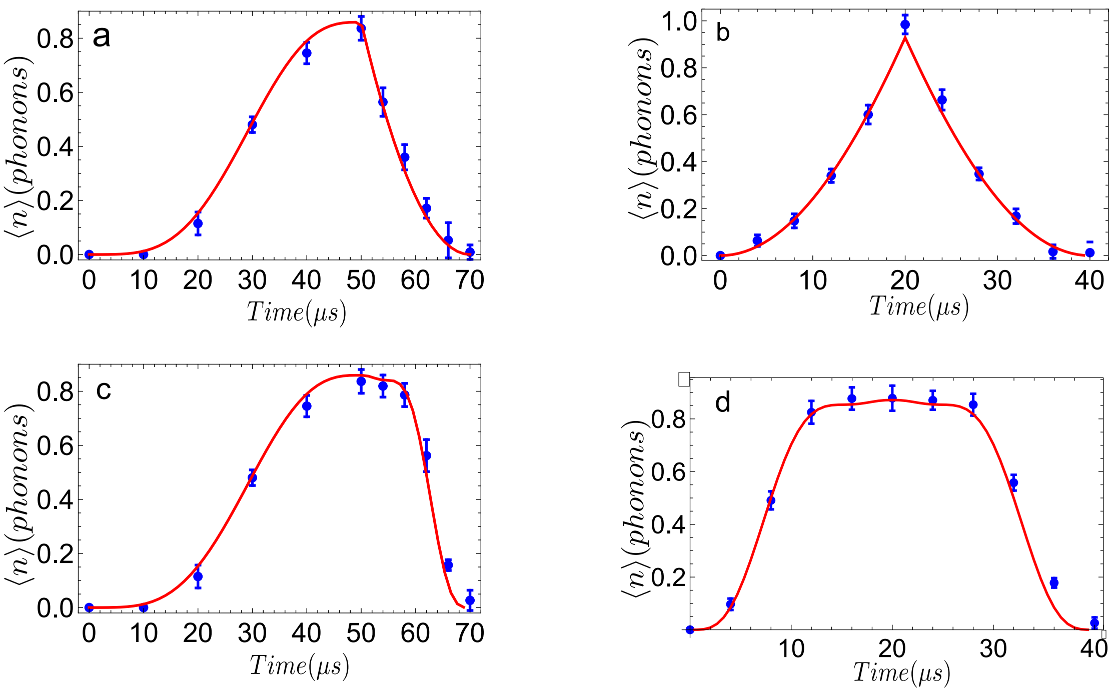

Supplementary Figure 4: **The excitations in the lab framework for (a,b) counterdiabatic driving and (c,d) the unitarily equivalent driving.** (a) We use the linear ramp to push out and the counterdiabatic transport to bring back. In the main paper, we stop and add another counterdiabatic transport to bring the population in the instantaneous framework back to the lab framework for the measurement. Here we stop at a specific time and directly apply blue sideband transitions to measure the excited average phonon in the lab framework. (b) We use only the counterdiabatic driving for the pushing out and bringing back processes. We stop at a specific time and measure the excited phonon in the lab framework. (c) We use the linear ramp to push out and the unitarily equivalent transport to bring back. In the main paper, we stop and add another counterdiabatic transport to bring the population in the instantaneous framework back to the lab framework for the measurement. Here we stop at a specific time and directly apply the blue sideband transition to measure the excited average phonon in the lab framework. Here the shortcut ratio is 0.4. (d) We use only the unitarily equivalent driving for the pushing out and bringing back processes. We stop at a specific time and measure the excited phonon in the lab framework. The error bars represent the standard deviation of 200 measurements of the average phonon excitation.

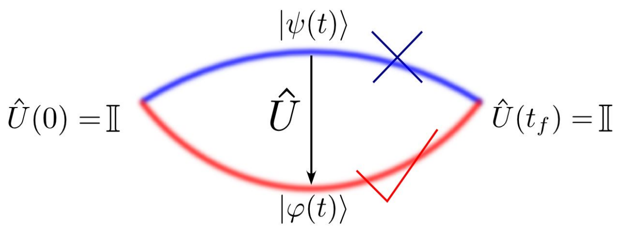

Supplementary Figure 5: **The UE protocol.** The transformation $\hat{U}(t)$ is used to find a UE Hamiltonian for the full CD Hamiltonian. In the UE protocol, the STA transport can be assisted exclusively with a local potential. For the modified dynamics to connect the same initial and final states, we impose the zeroth order boundary conditions $\hat{U}(0) = \hat{U}(t_f) = \mathbb{I}$ and the first order boundary conditions $\dot{f}(0) = \dot{f}(t_f) = 0$.

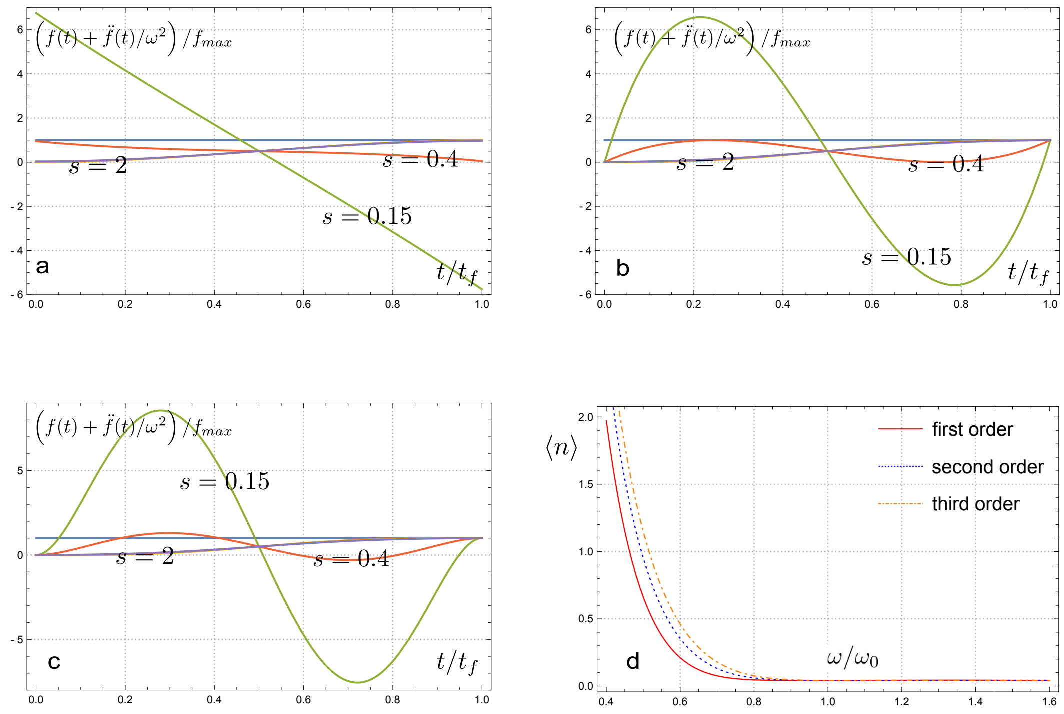

Supplementary Figure 6: **Waveforms of (a) the first, (b) second and (c) third order polynomials and (d) their robustness against trap frequency errors.** In (d), the robustness for different solutions against the trap frequency errors is compared for the case of $s = 1$.

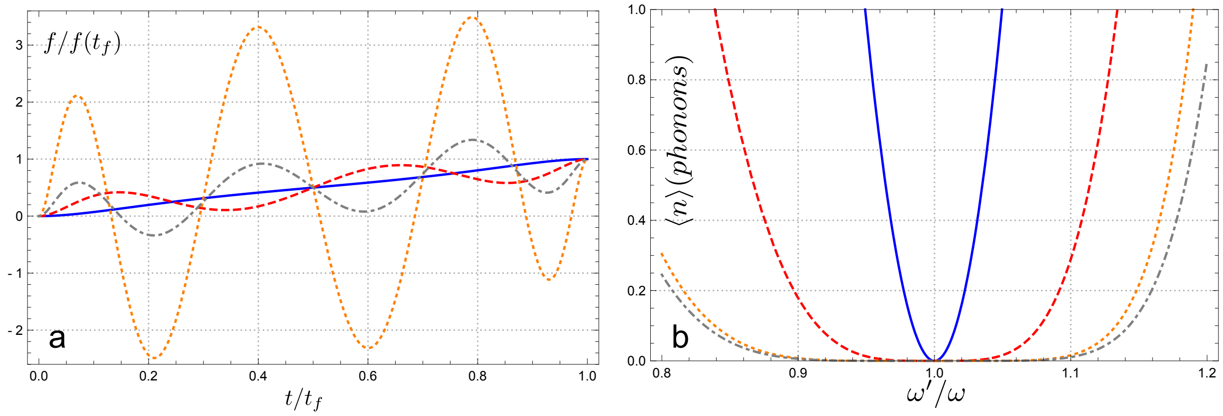

Supplementary Figure 7: Waveforms for the Fourier optimization scheme of different order N and their robustness against the trap frequency errors. (a) The maximum amplitude of $f(t)$ increases rapidly with the order N and decreases quickly with the shortcut ratio s. (b) With the same $s = 1$, the robustness would be better for a larger order N. With the same order $N = 3$, the robustness would be better for a larger shortcut ratio s.

SUPPLEMENTARY NOTE 1. DRAGGED HARMONIC OSCILLATOR MODEL

In this section we provide a microscopic derivation of the dragged harmonic oscillator model. We utilize one of the transverse motional modes of a single $^{171}\mathrm{Yb}^+$ ion trapped in the linear four rod Paul trap. The energy levels and all the definitions of symbols are shown in Fig. 1 of the main text.

The internal state of the ion is $c_g(t)|g\rangle + c_e(t)|e\rangle$. We define the slow varying amplitude $C_i(t) \equiv c_i(t)e^{i\omega_i t}$ ($i = g,\ e$), where ω_i is the energy of the corresponding internal state. Using the dipole approximation we can write the equation of motion

$$i\dot{C}_g(t) = \frac{C_e(t)}{2}\left(g_b e^{-i(\vec{k}_b\cdot\vec{x}+\Delta t)} + g_r e^{-i(\vec{k}_r\cdot\vec{x}+(\Delta+\delta)t-\phi)}\right), \tag{1}$$

$$i\dot{C}_e(t) = \frac{C_g(t)}{2}\left(g_b^* e^{i(\vec{k}_b\cdot\vec{x}+\Delta t)} + g_r^* e^{i(\vec{k}_r\cdot\vec{x}+(\Delta+\delta)t-\phi)}\right). \tag{2}$$

In the rotation wave approximation (RWA), we adiabatically eliminate the excited state $|e\rangle$ and get the equation of motion for the ground state $|g\rangle$ only

$$i\dot{C}_g(t) = -\Omega C_g(t) - \frac{\Omega}{2}\left(e^{i(\Delta\vec{k}\cdot\vec{x}+\delta t-\phi)} + H.c.\right)C_g(t). \tag{3}$$

In the right hand side of the equation, the first term $-\Omega = -\frac{g_b g_r^\star}{2\Delta}$ before C_g is the AC Stark shift and $\delta\vec{k} = \vec{k}_r - \vec{k}_b$ and $\phi = \phi_r - \phi_b$. If we neglect the AC Stark shift and only consider one of the motional modes (others are ignored by the RWA) in the Lamb-Dicke regime, we find the effective Hamiltonian for the ground state

$$\hat{H}_{eff} = \frac{\Omega\Delta k}{2}x_0\left(\hat{a}e^{-i(\omega t+\phi)} + H.c.\right). \tag{4}$$

Here a global phase of $-\pi/2$ has been added. In this experiment, Ω, ϕ and ω can be changed to control the amplitude of the force term $f(t)\hat{x}$, realize the momentum term $h(t)\hat{p}$ and vary the nominal trap frequency. As a result, the dragged harmonic oscillator can be fully controlled.

In Supplementary Figure 1, we illustrate different transport protocols that we experimentally study. Sufficiently slow driving corresponds to the adiabatic transport. Nonadiabatic excitations appear when the protocol is sped up. To suppress them a global interaction according to the CD protocol is applied. As an alternative to this nonlocal interaction, we consider a unitarily equivalent protocol where the dynamics is assisted by a local potential so as to mimic adiabaticity in the preparation of the final target state. A variant of this later

protocol relies on the the Fourier optimization scheme, whereby the time-dependent force is designed such that the Fourier transform of the acceleration of the displacement vanishes when the angular frequency matches the trap frequency. The resulting protocol involves an oscillatory trajectory, see Section F for further details. All these protocols can be realized in the dragged harmonic oscillator model.

SUPPLEMENTARY NOTE 2. PERFORMANCE OF STA DEPENDING ON THE SHORTCUT RATIO

To test the STA's performance against the shortcut ratio s, we measure the final phonon excitation after the CD backward process with different shortcut ratios as shown in Supplementary Figure 2(a). We do not observe any meaningful excitation in phonon number after the CD protocol from $s = 0.15$ to $s = 1$.

We also test the final excitations against different shortcut ratios from $s = 0.4$ to $s = 1.0$ for the UE protocol as shown in Supplementary Figure 2(b). For the maximum laser intensity, the minimum shortcut ratio for the UE protocol is 0.4 in our experimental condition.

SUPPLEMENTARY NOTE 3. MEASUREMENT OF DYNAMICS IN THE INSTANTANEOUS BASIS

In order to measure the phonon excitation in the instantaneous basis during the transport, we stop the protocol during the process and adiabatically bring back the phonon system to the lab framework by performing a CD with $s = 0.15$ as shown in Supplementary Figure 3.

SUPPLEMENTARY NOTE 4. THE EXCITATION OF PHONONS DURING THE TRANSPORT IN THE LAB FRAME

Additional data for the tests of the CD and the UE protocol are reported in Supplementary Figure 4. Instead of the excitation in the instantaneous framework, we also measure the excited average phonon number in the lab framework. The data confirm that we actually transport the ion in the lab framework.

From Supplementary Figure 4, we also find the UE will excite more phonon than the

CD in the lab framework. Because the higher phonon states are more fragile [1], we expect the CD will be more robust against the heating effect. This can be the reason for the tiny difference between the final excitations showed in Supplementary Figure 2.

SUPPLEMENTARY NOTE 5. THE WAVEFORMS FOR DIFFERENT UE TRANSPORTS

Here we provide waveforms for different UE transports. In order to remove the non-local control term proportional to $\hat{p}$ in the CD protocol, a momentum shift transformation $\hat{U}(t)$ is applied. As shown in Supplementary Figure 5, it is necessary to fulfill the zeroth and the first boundary conditions for $f(t)$, which are $\hat{U}(0) = \hat{U}(t_f) = \mathbb{I}$ and $\dot{f}(0) = \dot{f}(t_f) = 0$, respectively. However, it is not the sufficient condition. We can impose higher-order boundary conditions for the transports. Supplementary Figure 6(a)-(c) summarizes the waveforms for different orders of polynomial solutions with different shortcut ratios.

Supplementary Figure 6(d) shows the comparison of the robustness against the trap frequency errors for the case of $s = 1$. The robustness of the first order waveform is the best, which is consistent to the result in Ref. [2]. However, it requires the instantaneous switching of the control field, which is difficult in our experimental system. Therefore, we use the second order polynomial waveform in the experiment.

SUPPLEMENTARY NOTE 6. FOURIER OPTIMIZATION SCHEME

In this section we briefly describe the STA protocol based on the Fourier optimization scheme, which is well-explained in Ref. [3] for the self-consistency of the manuscript. The method exploits the fact that when a force $f(t)$ is applied to a particle in the ground state from $f(0) = 0$ to $f(t_f)$ with the first order boundary conditions $\dot{f}(0) = \dot{f}(t_f) = 0$, the final excitation energy is written by

$$\Delta E(\omega_0) = \left| \int_0^{t_f} \ddot{f}(t') e^{-i\omega t'} dt' \right| / m\omega^2, \tag{5}$$

where ω is the trap frequency. If we'd like to optimize the protocol for two-different trap frequencies ω_1, ω_{21}, we have to minimize the excitation $\Delta E(\omega_1)$ and $\Delta E(\omega_2)$ with proper $f(t)$. This is the principle to design the two-frequency transport waveform. If we set $\omega_1 = \omega_2 = \omega$,

the robustness of the protocol against the trap frequency error is increased. In Ref. [3], an auxiliary function $g(t)$ is introduced to satisfy the following condition,

$$\ddot{f}(t) = \frac{d^4 g}{dt^4} + (\omega_1^2 + \omega_2^2)\frac{d^2 g}{dt^2} + \omega_1^2\omega_2^2 g(t), \tag{6}$$

with the boundary conditions $g(0) = g(t_f) = \dot{g}(0) = \dot{g}(t_f) = \ddot{g}(0) = \ddot{g}(t_f) = \dddot{g}(0) = \dddot{g}(t_f) = 0$. Combining Eqs. (5) and (6), we get

$$\Delta E(\omega) = \left|(\omega^2 - \omega_1^2)(\omega^2 - \omega_2^2)\int_0^{t_f} e^{-i\omega t'} g(t')dt'\right| \tag{7}$$

and find $\Delta E(\omega_1) = \Delta E(\omega_2) = 0$. Therefore, we can choose a polynomial function $g(t)$ to satisfy the boundary conditions for $g(t)$ as well as $f(t)$. For this kind of the order $N = 2$, we can choose $g(t) \propto (t/t_f)^4(1 - t/t_f)^4(1 - 2t/t_f)$. The technique can be generalized for the multiple frequency cases by satisfying the condition of $\Delta E(\omega_i) = 0$ for a set of frequencies $\{\omega_i | i = 1, \ldots, N\}$. By setting these frequencies to be degenerate, the protocol exhibits an enhanced robustness against frequency errors with the order N. For the N-order case $g(t) \propto (t/t_f)^{2N}(1 - t/t_f)^{2N}(1 - 2t/t_f)$, from which one can derive $f(t)$. In the main paper we show the waveform of order $N = 3$ for $s = 1.5$ due to the limitation of $f(t)/f_{max} < 1$. In Supplementary Figure 7 we compare waveforms of different values of N and their robustness against the trap frequency errors. We can see the maximum amplitude of the control field of this protocol is extremely sensitive to N and s. And for a fixed protocol duration a higher robustness can be achieved by increasing the amplitude of the control field.

SUPPLEMENTARY REFERENCES

[1] Q. A. Turchette, C. J. Myatt, B. E. King, C. A. Sackett, D. Kielpinski, W. M. Itano, C. Monroe, and D. J. Wineland, Phys. Rev. A **62**, 053807 (2000).

[2] H.-K. Lau and D. F. V. James, Phys. Rev. A **83**, 062330 (2011).

[3] D. Guéry-Odelin and J. G. Muga, Phys. Rev. A **90**, 063425 (2014).
